# New mouse genetic model of breast cancer from *IKKα* defects in dendritic cells revealed by single-cell RNA sequencing

**DOI:** 10.1038/s41421-023-00553-z

**Published:** 2023-07-18

**Authors:** Weiwei Lai, Wanshan Hu, Yinming Liang, Lifang Yang, Chao Mao, Tania Tao, Xiang Wang, Desheng Xiao, Shuang Liu, Yongguang Tao

**Affiliations:** 1 NHC Key Laboratory of Carcinogenesis, Cancer Research Institute and School of Basic Medicine, Department of Pathology, Xiangya Hospital, Key Laboratory of Carcinogenesis and Cancer Invasion, Central South University, Changsha, Hunan China; 2 Laboratory of Genetic Regulators in the Immune System, School of Laboratory Medicine, Xinxiang Medical University, Xinxiang, Henan China; 3 Department of Thoracic Surgery, Hunan Key Laboratory of Tumor Models and Individualized Medicine, Second Xiangya Hospital, Central South University, Changsha, Hunan China; 4 Department of Oncology, Institute of Medical Sciences, National Clinical Research Center for Geriatric Disorders, Xiangya Hospital, Central South University, Changsha, Hunan China

**Keywords:** Breast cancer, Cancer of unknown primary

Dear Editor,

Breast cancer accounts for 12% of all new annual cancer cases and has become the most common cancer among adult malignancies^1^. The HER2-targeted therapy has significantly improved breast cancer treatment^2^. MMTV-neu and MMTV-PyMT mice have been extensively studied as models for breast cancer research. IκB kinase α (IKKα), one of the two catalytic subunits of the classical IKK complex that belongs to the upstream of NF-κB signaling, plays a critical role in carcinogenesis, including the development of lung adenocarcinoma with *KRAS* mutation^3,4^. Deficiency of *IKKβ*, another subunit of the IKK complex, results in impaired dendritic cell (DC) migration and immune tolerance^5^. Interestingly, we found that the conditional knockout of *IKKα* in mouse DCs (*IKKα*^*△Itgax*^) unexpectedly induced spontaneous tumors, which appeared near the mammary gland, with ~39.6% incidence of tumors in aged mice (≥ 20 weeks old, 23/58), and the spleen weight was increased with frequent splenomegaly (Supplementary Fig. S1). Furthermore, we identified that *IKKα* was indeed knocked out in the bone marrow-derived DCs of *IKKα*^*△Itgax*^ mice, and the IKKα level was normal in several other tissues (Supplementary Fig. S2). However, the pathological type and origin of this spontaneous tumor are not clear to us, and the reliability of the mouse model for exploring the breast cancer immune microenvironment remains to be tested as well.

Recently, single-cell RNA sequencing (scRNA-seq) has been widely used to identify sources of malignant cells and tumor types^6,7^. Consequently, we applied scRNA-seq to systematically identify this unknown tumor type. We also downloaded 11 tissue scRNA-seq datasets from the Tabula Muris database^8^ (Supplementary Fig. S3a, b). We then performed PCA analysis and found that normal mammary tissues and tumor tissues had similar gene expression patterns and shared similar immune phenotypes (Supplementary Fig. S3c, d), indicating that the tumor of *IKKα*^*△Itgax*^ mice might originate from the mammary tissue.

To further identify the mammary-like properties of tumors in *IKKα*^*△Itgax*^ mice, we detected 12 clusters of cells by an unsupervised method, and each cluster was annotated by well-known markers to classify those cells (Fig. 1a). UMAP plots identified that *Erbb2* and *Vimentin* were enriched in epithelial cells (Supplementary Fig. S3e). To determine genes driving the tumors in *IKKα*^*△Itgax*^ mice, we identified the differentially expressed genes (DEGs) by comparing different clusters of tumor and normal mammary cells. We found that *S100a8*, *Erbb2*, and *Tnfrsf1b* were enriched in the tumor epithelial cells compared to normal mammary cells (Supplementary Fig. S4a). To examine the functional implications of gene signatures, we performed the single-sample gene set enrichment analysis (ssGSEA) between the tumor cells and normal mammary cells^9^. The targeting Kras and glycolysis signalings were upregulated in the tumor epithelial cells compared with the normal mammary tissue (Supplementary Fig. S4b), suggesting that the *IKKα* deficiency-induced spontaneous tumors may originate from the epithelial cells.

**Fig. 1:**
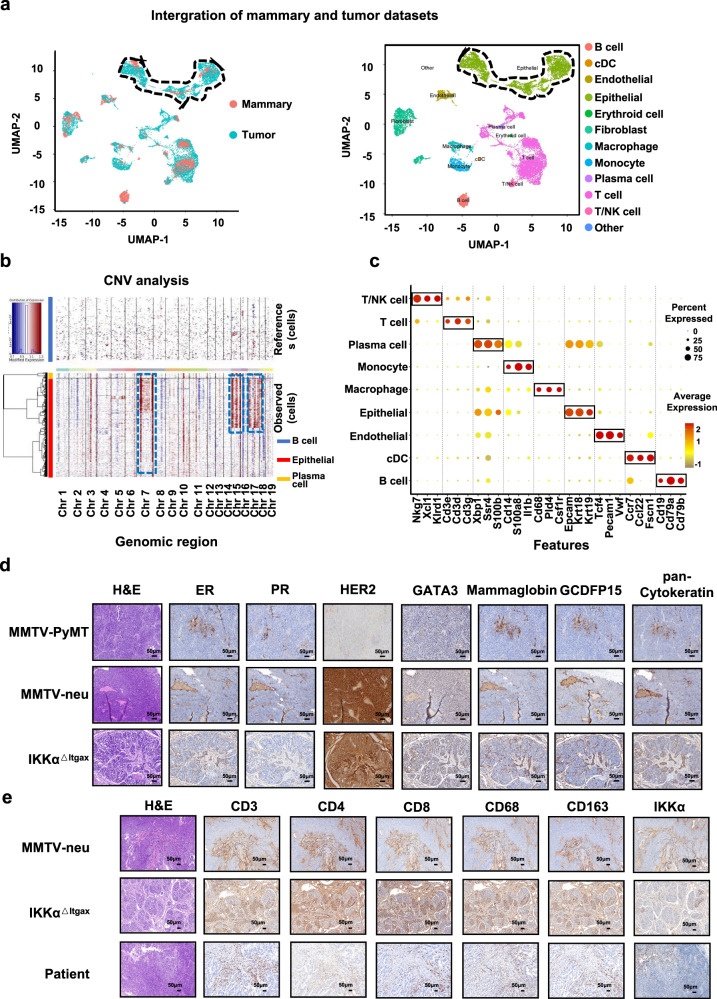
Identification of mouse tumor pathology and origin through scRNA-Seq and IHC. **a** UMAP plots of the 5362 mammary cells and 25,312 tumor cells colored by samples (left). UMAP plots of the mammary and tumor cells by clusters, color-coded by cell subsets as indicated (right). **b** The landscape of inferred large-scale CNVs for epithelium from the tumor samples. B cells were used as reference cells. The annotation tracks on the left indicate the corresponding cells: plasma cells (yellow), epithelial cells (red), and B cells (blue). Chromosome numbers are labeled at the bottom. **c** Dot plot of differentially expressed key cell-type marker genes in different cell clusters. **d** Hematoxylin & Eosin (H&E) staining and IHC show the expression of genes in the tumors of MMTV-neu, MMTV-Pymt, and *IKKα*^*△Itgax*^ mice. **e** H&E staining and IHC show the expression of IKKα and immune cell markers in MMTV-neu mice, *IKKα*^*△Itgax*^ mice, and human breast cancer cells (*Her2*-positive).

Previous evidence suggested that epithelial cells have differentiation states and gene expression patterns of tumor cells from precancerous to malignant states by trajectory analysis, which can be used to study the molecular characteristics of carcinogenesis^10,11^. We then constructed a transcriptional trajectory of the tumor cell and normal epithelial cells. The tumor epithelial samples formed a branched structure with five transcriptional states (S1–S5) along the normal epithelial cells, marking their distinct differentiation states, whereas the normal epithelial cells were mostly located in S1 (Supplementary Fig. S4c, d). Large-scale copy number variations (CNVs) were used to infer this tumor cell source, and we found that 7q, 15q, 17q, and 18q gains as unique events were present in the tumor epithelial cells (Fig. 1b). We also examined the pathological types in the two common breast cancer mouse models and our spontaneous tumor model, and demonstrated that the spontaneous tumor has characteristics of breast cancer with high levels of Her2, Gata3, and cytokeratin (Fig. 1d). To further investigate the immunophenotype of breast cancer patients and breast cancer mouse models, we detected the expression of IKKα and immune cell markers by immunohistochemistry (IHC), and found that IKKα was mainly expressed in the cytoplasm (Fig. 1e). Quantified CD8^+^/CD4^+^ cell ratio was higher in *IKKα*^*△Itgax*^ mice and breast cancer patients compared with MMTV-neu mice, whereas the CD163^+^/CD68^+^ cell ratio was decreased in *IKKα*^*△Itgax*^ mice and breast cancer patients (Supplementary Fig. S5a–c). Moreover, TCGA data showed that *IKKα* levels were correlated with *HER2* expression in breast cancer (Supplementary Fig. S5d). Taken together, these data suggested that our mouse model tumor has breast cancer features and is more suitable for mimicking the tumor microenvironment of breast cancer.

To further examine the immune cells of the tumor microenvironment, particularly the DCs and T cells, we evaluated the lineage-specific gene expression features of cell clusters by DEG analysis and found that the classical dendritic cell (cDC) like population has increased expression of *Ccr7*, *Ccl22*, and *Fscn1* (Fig. 1c). Genes encoding Relb, Pten, Tcf4, and Irf7 were downregulated in the tumor DCs (Supplementary Fig. S6a), suggesting that the tumor DCs have impaired DC development compared with normal mammary DCs. We also found that antigen processing and presentation and T-cell activation signaling were downregulated in the tumor DCs compared with normal mammary DCs (Supplementary Fig. S6b). The expression of *Tcf4* was decreased in T cells from the tumors compared with that in the normal mammary tissue (Supplementary Fig. S6c). The apoptosis signaling was upregulated in T cells from the tumors, while the activated T-cell proliferation and adaptive immune response signalings were downregulated in the tumor T cells compared with those in the normal mammary T cells (Supplementary Fig. S6d), suggesting that *IKKα* deficiency in the DCs impaired T-cell activation. We also ordered T cells from the normal mammary tissues and the tumor samples in a pseudo-temporal manner using Monocle and generated trajectory plots. T cells from the tumors were located in the pre-branch and bifurcated into two diverse branches (Supplementary Fig. S6e). Most normal mammary T cells were present in the distinct branch S3 (Supplementary Fig. S6f). These data further indicated that loss of *IKKα* in the DCs induced T-cell dysfunction, thus contributing to tumor immune evasion.

To further identify the dendritic Cell and T-Cell Interaction (CCI), we calculated the CCI scores that represent the communication probabilities among all pairs of the subclusters across all ligand–receptor pairs^12^. A stronger CCI was observed in normal mammary samples than that in the tumor samples, and the *Ccl*, *Cd86*, *Faslg*, and *Ifn* were strongly upregulated in the mammary DCs (Supplementary Fig. S7a, b). Similar patterns were observed in the DCs with outgoing or incoming interactions with T cells (Supplementary Fig. S7c, d). We also performed flow cytometry to investigate monocytes, granulocytes, and natural killer cells in *IKKα*^*△Itgax*^ mice of different ages. The percentage of monocytes was decreased both in the blood and spleen and was significantly reduced in the lungs of aged *IKKα*^*△Itgax*^ mice (20 weeks old) compared with wild-type (WT) mice (Supplementary Fig. S8a–d). We also found that the proportion of granulocytes significantly increased in the peripheral blood and lungs from both young and aged *IKKα*^*△Itgax*^ mice compared with WT mice (Supplementary Fig. S8e–g). Moreover, *IKKα* deficiency in the DCs of aged mice decreased natural killer cells in the peripheral blood and lung. However, there was no significant difference in the number of natural killer cells in the spleen of *IKKα*^*△Itgax*^ mice compared with WT mice (Supplementary Fig. S8h–j). These data revealed that loss of *IKKα* in DCs affects cell populations, which may be associated with the imbalance of immune homeostasis.

To gain insights into the tumor-infiltrating immune cells between the *IKKα*^*△Itgax*^ tumors and the MMTV-neu tumors (GEO: GSE122336)^13^, we sorted CD45^+^ cells from the scRNA-seq data and obtained six clusters (Supplementary Fig. S9a). Although these two groups share similar immune features, a decreased macrophage population, and an increased T-cell infiltration were observed in the *IKKα*^*△Itgax*^ tumor. Moreover, the *Pd1* and *Ctla-4* were upregulated in the *IKKα*^*△Itgax*^ tumor-infiltrating T cells (Supplementary Fig. S9b, c). The UMAP plots identified a strong expression of *Ccr7*, *Ccl22*, and *Cd68* characteristic of DCs (Supplementary Fig. S9d). The monocle pseudotime analysis revealed that T cells from MMTV-neu mice were present in the late cell states, and macrophages in *IKKα*^*△Itgax*^ mice exhibited an original cell differentiation state (Supplementary Fig. S10). These results further substantiated the fact that the *IKKα*^*△Itgax*^ tumor infiltration T cells and macrophages consist of primary cell states compared to those in MMTV-neu mice, which may contribute to tumor immune escape.

To the best of our knowledge, it is the first case showing that genetic defects in immune cells induced a solid tumor. We propose that it is worthwhile to identify whether genetic mutations in DCs epigenetically regulate breast epithelial cells and ultimately cause epithelial carcinogenesis. New discoveries may lead to new strategies for tumor prevention and treatment. Future studies are needed to clarify the molecular mechanism of this novel breast cancer model, as well as the specific tumor microenvironment characteristics, and determine its specific time period of mammary cell hyperplasia and the sensitivity to Her2-targeted therapy in the *IKKα*^*△Itgax*^ mice. We also believe that our research will provide a novel mouse model for exploring immune therapy for breast cancer and facilitate vaccine development against breast cancer through promoting IKKα signaling in DCs.

## Supplementary information

Ethical approval

Supplementary file

## References

[1] Sung H (2021). CA Cancer J. Clin..

[2] Chan A (2021). Clin. Breast Cancer.

[3] Xiao D (2015). Oncotarget.

[4] Vreka M (2018). Cancer Res..

[5] Baratin M (2015). Immunity.

[6] Wang R (2021). Nat. Med..

[7] Hu L (2021). Cancer Res..

[8] Nicholas S (2018). Nature.

[9] Shen S (2019). EBioMedicine.

[10] Peng J (2019). Cell Res..

[11] Kim N (2020). Nat. Commun..

[12] Jin S (2021). Nat. Commun..

[13] Wang Q (2019). Nat. Commun..

