# Supplementary material for: New mouse genetic model of breast cancer from IKKα defects in dendritic cells revealed by single-cell RNA sequencing
Source: Cell Discov. 2023 Jul 18;9:72. doi: 10.1038/s41421-023-00553-z (PMC10352231; doi:10.1038/s41421-023-00553-z)
Supplement: Supplementary file 1 — Ethical approval [file 41421_2023_553_MOESM1_ESM.pdf]

中南大学湘雅医院生物样本的收集知情同意书

研究项目名称：采用单细胞测序鉴定 DC 特异性敲除 IKKα转基因小鼠自发肿瘤来源的研究

研究方：中南大学湘雅医院病理科，中南大学基础医学院肿瘤研究所

方案版本号日期：2021 年 2 月 28 日

知情同意书版本号日期：2021 年 2 月 28 日

主要研究者：赖巍巍

******知情部分

尊敬的先生/女士：我们诚邀您参加中南大学湘雅医院病理科和基础医学院肿瘤研究所发起的一项**“采用单细胞测序鉴定 DC 特异性敲除 IKKα转基因小鼠自发肿瘤来源的研究”**生物样本收集的临床研究项目。我们需要您捐赠(提供)您在乳腺癌切除手术的部分组织给我们作为临床科研用。组织样本的收集是在您的诊断和治疗过程中必须进行切除或穿刺后所取得的活组织,且是在标本离体后，在充分保证病理诊断所需之后才留取部分组织。这些样本的收集将会被用于与肿瘤治疗相关的生物医药研究、寻找有助于判预后好坏的因子,可能包括一些基因表达研究和遗传学研究等。这些研究可能将有助于为未来临床选择最为合适的治疗方案或为药物治疗效果提供预测等。这些研究所收集的样本将在低温下保存，样本的保存地点在中南大学湘雅医院病理科，保存时限是半年，至时限时我们将按照国家的相关规定规范销毁样本。样本是用于对人类健康有益的研究项目,我们将遵照国家的严格的审查程序及相关规定使用样本，以保证科研的合理性和可行性,并符合伦理法律规范。我们的样本收集是公益性、非盈利性的，本身是没有获益的，对您本人也没有经济获益。但未来研究的结果有可能会为您以及与您相似的患者的疾病提示新的治疗和诊断方法,从而给您和与您类似的其他患者带来益处。当然，您具有充分的隐私权。样本和所有信息的采集都将在法律允许的范围内实现全面的保密,您的身份识别仅为样本编码或研究编码。在任何研究报告和出版物中您将不会被辨认出来。您是否愿意提供样本是完全自愿的，邀请您参加是期待本项目能为后来的患者带来更多诊断和治疗疾病的方法。当然如果您选择不参加或选择在任何时间退出样本提供，这都不会对您的医疗待遇与权益受到任何不良影响。按国家相关规定，本研究信息将在国家医学研究备案系统 http://114.255.48.20 备案，您可以登录该网站了解研究相关信息（不含有个人信息）。

感谢您的配合！

如果您有任何关于项目的疑问请拨打研究者电话:13549669554

如果您有任何关于权益的疑问请拨打伦理委员会电话:0731-84327919

******同意部分

1.我已经阅读并理解了本知情同意书的全部内容。

2.我有机会提问而且所有问题均已得到解答。

3.我理解参加本研究完全是自愿的,我也可以选择在任何时候退出这一活动，我的任何权益不会因此而受到影响。

4.我☑希望☐不希望被告知任何与我的健康有重大关联的研究结果。

5.我清楚签署以后还有疑问可以咨询伦理委员会的工作人员。(电话: 0731-84327919)

6.我会收到一份经过签名并注明日期的知情同意书副本 。

7.我同意捐赠样本和信息以供未来科学研究,并同意伦理委员会审核使用本人样本和信息的适当性。

捐赠者签名: 刘伟利　　电话:15343911108日期:2022年 8月 16日

(注:如果捐赠者无行为能力/限制行为能力时,则需监护人签名和签署日期)

捐赠者的监护人签名:　　电话:　　日期:　　年　月　日

与捐赠者关系

研究者签名: [illegible]　　电话: 13549669554 日期:2021年 4月 16日

研究者指定的研究人员姓名(印刷体)：赖巍巍
研究者指定的研究人员签名(手写体)：[illegible]
电话:　13549669554　　日期: 2021 年 3 月 18 日
