# Supplementary material for: New mouse genetic model of breast cancer from IKKα defects in dendritic cells revealed by single-cell RNA sequencing
Source: Cell Discov. 2023 Jul 18;9:72. doi: 10.1038/s41421-023-00553-z (PMC10352231; doi:10.1038/s41421-023-00553-z)
Supplement: Supplementary file 2 — Supplementary file [file 41421_2023_553_MOESM2_ESM.pdf]

Supplementary materials for

New mouse genetic model of breast cancer from IKKα defects in dendritic cells revealed by single-cell RNA sequencing

Weiwei Lai [1], Wanshan Hu [1], Yinming Liang [2,3], Lifang Yang [1], Chao Mao [1], Tania Tao [1], Xiang Wang [5], Desheng Xiao [1*], Shuang Liu [4*], and Yongguang Tao [1,4*]

1. NHC Key Laboratory of Carcinogenesis, Cancer Research Institute and School of Basic Medicine, Department of Pathology, Xiangya Hospital, Key Laboratory of Carcinogenesis and Cancer Invasion (Central South University, Ministry of Education), Central South University, Hunan, 410078 China
2. Henan Collaborative Innovation Center of Molecular Diagnosis and Laboratory Medicine, School of Laboratory Medicine, Xinxiang Medical University, Xinxiang, Henan, 453003, People's Republic of China
3. Laboratory of Genetic Regulators in the Immune System, School of Laboratory Medicine, Xinxiang Medical University, Xinxiang, Henan, 453003, People's Republic of China.
4. Department of Oncology, Institute of Medical Sciences, National Clinical Research Center for Geriatric Disorders, Xiangya Hospital, Central South University, Changsha, Hunan, 410008 China
5. Department of Thoracic Surgery, Hunan Key Laboratory of Tumor Models and Individualized medicine, Second Xiangya Hospital, Central South University, Changsha, 410011 China

* Corresponding authors. Y.T. Email: taoyong@csu.edu.cn, Department of Pathology, Xiangya Hospital, Central South University;
S.L. Email: shuangliu2016@csu.edu.cn, Department of Oncology, National Clinical Research Center for Geriatric Disorders, Xiangya Hospital, Central South University.
X.D. Email: xds96@csu.edu.cn, Department of Pathology, Xiangya Hospital, Central South University.

This file including material and methods, ethics approval, supplementary figure legends and figures.

Materials and Methods

Data Availability Statement

The single-cell data of normal mouse organs mentioned in this paper were obtained from public databases (http://tabula-muris.ds.czbiohub.org/). We also used single-cell sequencing data from immune cells in MMTV-neu tumors (GEO: GSE122336).

Preparation of single-cell suspensions

We prepared single-cell samples of mouse tumors according to the instructions of the Chromium Next GEM Single Cell 3' Reagent Kits v3.1 and BGI Genomics Co., Ltd to complete the subsequent sequencing work.

First, the three spontaneous tumor form IKKα$^{F/F}$ Itgax^{Cre} mice were prepared in single-cell suspension, and then the cell count and cell viability test were performed. If the cell viability was ≥80%, the cell concentration was adjusted to 700–1200 cells /μl. Using a microfluidic chip, the prepared cell suspension was coated with cell barcode gel beads and cells in the droplet. In the droplets, the cells rupture, and the released mRNA is linked to the cell tag sequences on the gel beads to form single-cell GEMs structures (Gel Bead in Emulsions). A reverse transcription reaction was performed in the droplet to form cDNA, followed by demulsification and cDNA library construction.

scRNA-seq data analysis and graphing

After the raw sequence data was obtained, we mapped the sequences of the mouse genome reference using the CellRanger (3.0.1) pipeline. The generated

gene-barcode matrixes were submitted to Scrublet to remove the potential doublets. Then the 10x data matrixes were imported into Seurat V3.0 R package (https://satijalab.org/seurat) to perform data filtration, sample integration, gene normalization, dimension reduction, and data visualization. All the samples, including breast samples from normal mice in public databases, were integrated as one object using the Seurat "IntegrateData" function. Cells with low feature counts (<200) and high percent of mitochondrial genes (>20%) were removed. Dimension reduction was conducted using Seurat "RunPCA" function. Then, the Uniform Manifold Approximation and Projection for Dimension Reduction (UMAP) was used to visualize single-cell clusters employing the top 30 principles components with the largest variance (resolution=0.6).

Based on the cell clustering results, the likelihood ratio statistic test was used to screen differentially expressed genes (DEGs) of each cluster using Seurat's Bimodal function. The genes were considered as DEGs when they had the expression that satisfied the adjusted $p<0.05$ (corrected P-value from T test using the Benjamini-Hochberg correction) and LogFoldChang>=0.25 compared to other clusters. Cluster-specific maker genes were chosen according to their significant up-regulation in one cluster but not in other clusters. DEGs between tumor-derived cells and tumor-adjacent normal lung-derived cells were detected with Seurat's “FindMarkers” function using the Wilcoxon Rank Sum test.

Annotation of each cluster

First, each cluster was annotated based on the expression of canonical marker

genes that we curated from the literature and Cellmarker database (http://biocc.hrbmu.edu.cn/CellMarker/). We also referred cluster specific DEGs that are known to be cell markers to verify the cell type.

Gene Set Variation Analysis

Gene Set Variation Analysis (GSVA) was identified using the GSVA and ClusterProfiler R packages. The lists of Gene Sets that satisfy the adjusted p-value<0.05 and LogFoldChange>=0.1 in a cluster were used before we chose representative Gene Set terms illustrated with ggplot2.

CNV inferred from scRNA-seq

Infer CNV (https://github.com/broadinstitute/inferCNV) was used to identify large-scale chromosomal copy number variations of potentially malignant cells using single-cell RNA-seq data. The expression intensity of 12,328 genes across the malignant cell genome was compared to annotated T cells. We used the cutoff=0.1 as the minimum threshold to judge whether the gene was mutated. The heatmap was generated to illustrate the gains and deletions of large segments of chromosomes in the malignant cell genome compared to the T cells in all the samples.

Immunohistochemistry staining

Tissue sectioning and immunohistochemistry (IHC) staining of formalin-fixed paraffin-embedded (FFPE) tumor samples were performed following standard protocols. All sections were 4 μm thick and deparaffinized using xylene and ethanol gradient. Antigen retrieval was performed in a high-pressure heat repair process

using citrate buffer at pH 6.0.

After endogenous peroxidase was blocked by incubating 10 min in 3% H_2O_2, the slides were incubated with primary antibodies followed by HRP-linked secondary antibodies and diaminobenzidine (DAB) staining. Counterstaining was done with hematoxylin. Slides were dehydrated with sequential ethanol washes for 1 min each, starting with 75%, then 80%, and finishing with a 100% ethanol wash. Antibodies and reagents used are listed as follows: ER (Kit-0012; MXB Biotechnologies, Fuzhou, China), PR (Kit-0013; MXB Biotechnologies), HER2 (Kit-0043; MXB Biotechnologies), GATA3 (MAB-0695; MXB Biotechnologies), Mammaglobin (MAB-0561; MXB Biotechnologies), GCDFP15 (MAB-0230; MXB Biotechnologies), pan-cytokeratin (MAB-0009; MXB Biotechnologies), IKKα (11930S, Cell signaling).

Cell pseudotime trajectory analysis

Monocle 3 (https://cole-trapnell-lab.github.io/monocle3) was used to construct the pseudotime trajectory. In detail, the “cell_data_set” was built from the Seurat object of all the tumor cells using the data slot of the integrated assay. Dimension reduction was carried out using the UMAP, “learn_graph,” and “order_cells” functions to establish the trajectory. Every single cell was projected on the tree and formed the trajectory by DDRThree.

Cell-cell communication analysis

According to the cellchat (http://www.cellchat.org/index_inner.html), the official standard parameters on mice of immune cells in the IKKα$^{F/F}$ Itgax^{Cre} mice tumor and

normal mammary were analyzed in intercellular communication.

Flow cytometry

Single-cell suspensions were blocked with anti-FcR (clone 2.4G2, a kind gift from Dr. Yinming Liang). For cell surface staining, the single-cell suspensions were incubated with the antibody cocktails for 30 min at 4 °C, and the samples were then detected with flow cytometry (BD FACSAria II). Data were analyzed using FlowJo software (version 10). CD3e (cat.no 557666), CD11b (cat.no 552850), CD49b (cat.no 553858), Ly6G (cat.no 562737), and CD19 (cat.no 551001) antibodies were purchased from BD Biosciences (Heidelberg, Germany).

Immunoblotting (IB) assay

Cells were collected and lysed with IP lysis buffer containing protease inhibitor cocktail. Protein concentrations were determined with a bicinchoninic acid assay (Thermo Fisher). To isolation of nuclear and cytoplasmic fractions, cells were resuspended in 200 μL of buffer A (10 mM HEPES, pH 7.9, 10 mM KCl, supplemented with protease inhibitors) for 15 min. The cytosolic fraction was released after adding 10% Nonidet P-40 (final concentration is 0.625%), and collected supernatants as cytosolic fraction by centrifugation at 10,000 g for 30 s. The nuclear pellets were washed twice with 1 mL of buffer A, and added with 50 μl of buffer B (buffer A containing 1% SDS). Next, the samples were boiled for 10 min, and the nuclear fraction was obtained by centrifugation at 14,000 g for 10 min at room temperature. IKKα was purchased from Cell signaling (11930S) and β-actin was purchased from Sigma (A5441).

Ethics approval and consent to participate

This study was reviewed and approved by the Ethics Committees of Central South University, 2021-XMSB-0100.

Supplementary figure legends and figure

Figure S1 Loss of IKKα in DCs promotes spontaneous tumor development.

a Individual mouse tumor growth curves of IKKα$^{\triangle Itgax}$ mice, each line represents a spontaneous tumor. **b** Body weight of WT (IKKα$^{fl/f}$l) and IKKα$^{\triangle Itgax}$ mice at different ages. **c** Image of mouse tissues from IKKα$^{fl/fl}$ (wild-type, WT) and IKKα$^{\triangle Itgax}$ mice, including thymus, lung, kidney, liver and spleen. **d** Spleen weight was measured in WT and IKKα$^{\triangle Itgax}$ mice. Statistical analyses were performed with Student's t-test; *p <0.05, **p < 0.01, and ***p < 0.001.

Figure S2. The expression of IKKα in BMDCs, tumor cells and different mouse tissues. a Bone marrow cells from WT and IKKα$^{\triangle Itgax}$ mice were stimulated with 15 ng/ml GM-CSF and 10 ng/ml IL-4 for 6 days to generate BMDCs. BMDCs were lysed, and the indicated proteins were analyzed using western blotting. **b** Western blot analysis of the indicated proteins in 3 spontaneous tumors and adjacent tissues from IKKα$^{\triangle Itgax}$ mice. N, normal (adjacent tissues); T, spontaneous tumor. **c** H&E staining and IHC analysis of IKKα protein levels in different tissues from WT (IKKα$^{fl/fl}$) and IKKα$^{\triangle Itgax}$ mice. **d** IHC staining score of IKKα expression in different tissues. Three independent experiments were performed, and the data are shown as the mean ± SD.

Figure S3 Single-cell transcriptome map of mammary and unknown tumor cell lineages.

a Schematic of sample collection for scRNA-seq. **b** Number of samples from scRNA-seq data, 3 tumors, and 11 different tissues from the Tabula Muris database. **C** The heatmap displays scaled gene expression patterns in mammary and tumor samples. **d** Percentage of different cell types in mammary and tumor samples. **e** UMAP plots of normalized marker expression from tumor and normal mammary samples.

Figure S4. Epithelial phenotypic landscape in tumor and normal mammary samples.

a Gene expression pattern of epithelial cells in tumor and normal mammary samples. **b** ssGSEA enrichment score of canonical pathways/genes in epithelial between tumor and normal mammary samples. **c** Unsupervised transcriptional trajectory of tumor and normal mammary epithelial cells from Monocle, colored by cell state and sample. **d** Relative proportion of cell subsets and tissue origins for each cell state as displayed in (c).

Figure S5. IHC staining scores of the indicated proteins in MMTV-neu, and IKKα$^{\triangle Itgax}$ mice and breast cancer patients.

a IHC staining score of CD3 expression in MMTV-neu, and IKKα$^{\triangle Itgax}$ mice and human breast cancer patients. Three independent experiments were performed, and the data are shown as the mean ± SD. **b** IHC staining score of the CD8/CD4 ratio in MMTV-neu, IKKα$^{\triangle Itgax}$ mice and human breast cancer patients. Three independent experiments were performed, and the data are shown as the mean ± SD. **c** IHC staining score of the CD163/CD68 ratio in MMTV-neu, IKKα$^{\triangle Itgax}$ mice and human breast cancer patients. Three independent experiments were performed, and the data are shown as the mean ± SD. **d** The relationship between IKKα and HER2 was analyzed in BRCA (TCGA data) by using the TIMER 2.0 database.

Figure S6. Gene expression pattern and ssGSEA enrichment score of cell clusters in tumor and normal mammary cells.

a Heatmap of cDC gene expression in tumor and normal mammary samples. Specific genes are represented by arrows. Each column represents a single cell. **b** Bar chart comparing the ssGSEA enrichment score of canonical pathways/gene sets of the cDC collection of the Molecular Signatures Database between tumor and normal mammary scRNA-Seq data. **c** Heatmap of T-cell gene expression in tumor and normal mammary samples. **d** ssGSEA of canonical pathways/genes in T cells between tumor and normal mammary scRNA-Seq data. **e** Trajectory reconstruction of T cells from tumor and normal mammary samples, colored by cell state and sample. **f** Relative percentage of T cells in each cell state between tumor and normal mammary samples.

Figure S7. Impaired dendritic cell and T-cell interaction in IKKα$^{\triangle Itgax}$ mice compared with normal mammary cells. **a** Network representing the difference in cell-cell interactions between tumor and mammary samples. **b** Heatmap of the cell-cell interaction contribution in dendritic cells as ligands and T cells as receptors; rows indicate the signaling pathways, and columns indicate the samples. **c** Heatmap of the interaction strength of outgoing signaling in T cells, T/NK cells, and cDCs from tumor and mammary samples. T cells (red), T/NK cells (blue), and cDCs (green). **d** Heatmap of incoming signaling patterns in T cells, T/NK cells, and cDCs from tumor and mammary samples.

Figure S8. Effect of DC-specific depletion of IKKα on granulocytes, monocytes, and NK cells in different tissues. a Representative flow cytometry gating strategies for granulocytes, monocytes, and NK cells. **b-d** Flow cytometry analysis of $CD3e^{-}$ $CD11b^{+}$ Ly6G granulocytes from different tissues showing the percentages in the blood (b), lung (c), and spleen (d) from mice of different ages. **e-g** Proportions of $CD3e^{-}$ $CD11b^{-}$ $Ly6G^{+}$ monocytes in different tissues from WT and $IKK\alpha^{\triangle Itgax}$ mice were analyzed using FACS: blood (e), lung (f), and spleen (g). **h-j** Proportions of $CD3e^{-}$ $CD49b^{+}$ $CD19^{+}$ NK cells in different tissues from WT and $IKK\alpha^{\triangle Itgax}$ mice were analyzed using FACS: blood (h), lung (i), and spleen (j). All results are presented as the means ± SEMs. Statistical analyses were performed with Student's t t-test; *$p < 0.05$, **$p < 0.01$, and ***$p < 0.001$.

Figure S9. Immune cells from two distinct tumors with overlapping features.

a UMAP plots of the 2,373 MMTV-neu and 4,903 IKKα$^{\triangle Itgax}$ tumor CD45^{+} cells colored by sample. MMTV-neu (red), IKKα$^{\triangle Itgax}$ (blue). UMAP plots of CD45^{+} cells colored by cluster. **b** Percentage of different cell types in MMTV-neu and IKKα$^{\triangle Itgax}$ samples. **c** Dot plots of gene expression in T cells from MMTV-neu and IKKα$^{\triangle Itgax}$ samples. **d** UMAP plots of immune cell clusters colored by the normalized log-transformed expression of the indicated genes.

Figure S10. Analysis of different tumor model T-cell and macrophage trajectories in pseudotime. a Pseudotime of T cells from MMTV-neu and IKKα$^{\triangle Itgax}$ tumors inferred by Monocle. Each point represents a single cell, colored by cell state and sample. **b** Relative proportion of cell subsets and tissue origins for each cell state as shown in (a). **c** Trajectory reconstruction of macrophages from different tumor models, colored by cell state and sample. **d** Relative percentage of macrophages in each cell state between MMTV-neu and IKKα$^{\triangle Itgax}$ tumors.

Figure S1

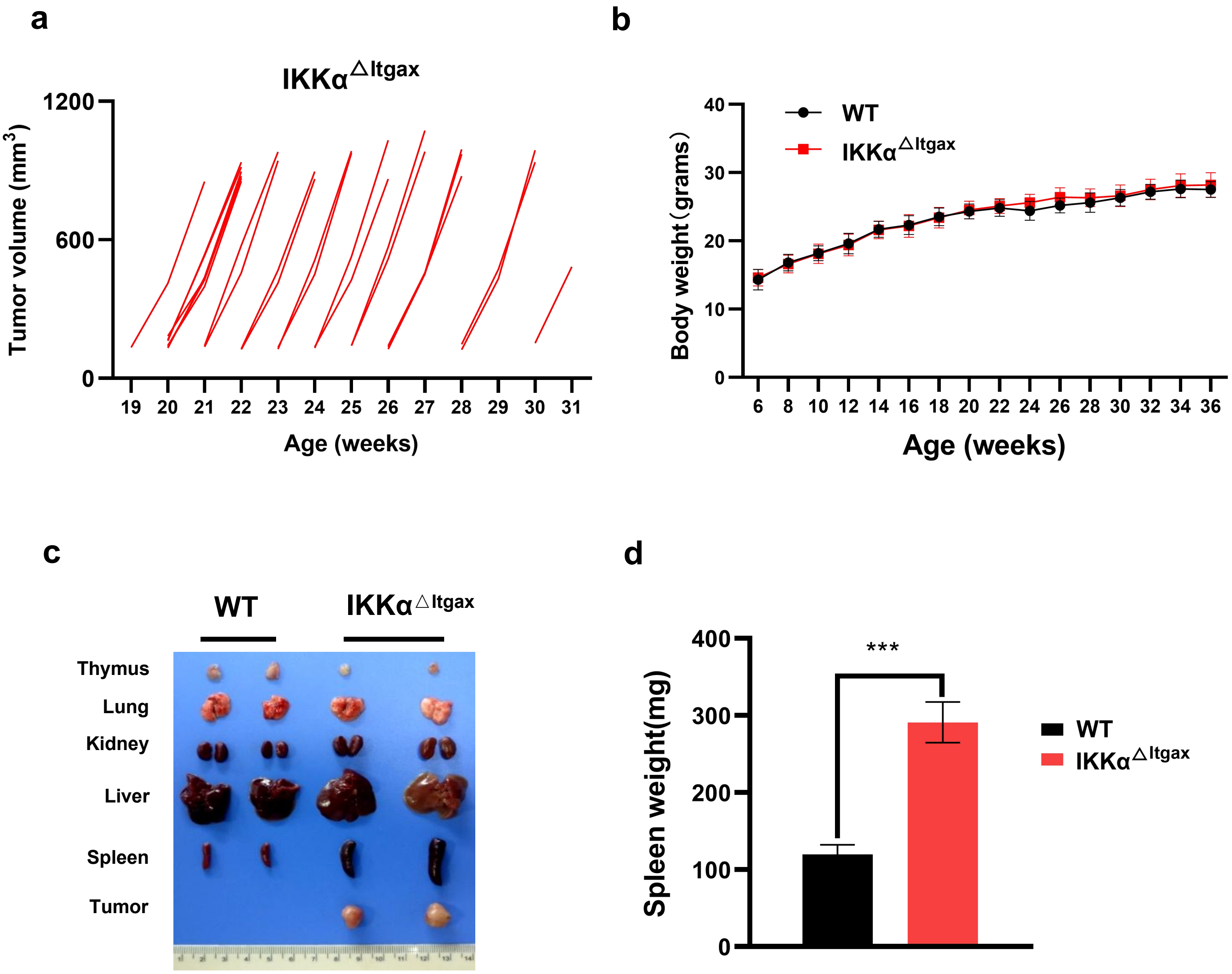
a
IKKα△Itgax
Tumor volume (mm3)
1200
600
0
19 20 21 22 23 24 25 26 27 28 29 30 31
Age (weeks)
b
Body weight（grams）
40
30
20
10
0
WT
IKKα△Itgax
6 8 10 12 14 16 18 20 22 24 26 28 30 32 34 36
Age (weeks)
c
WT
IKKα△Itgax
Thymus
Lung
Kidney
Liver
Spleen
Tumor
d
Spleen weight(mg)
400
300
200
100
0
***
WT
IKKα△Itgax

Figure S2

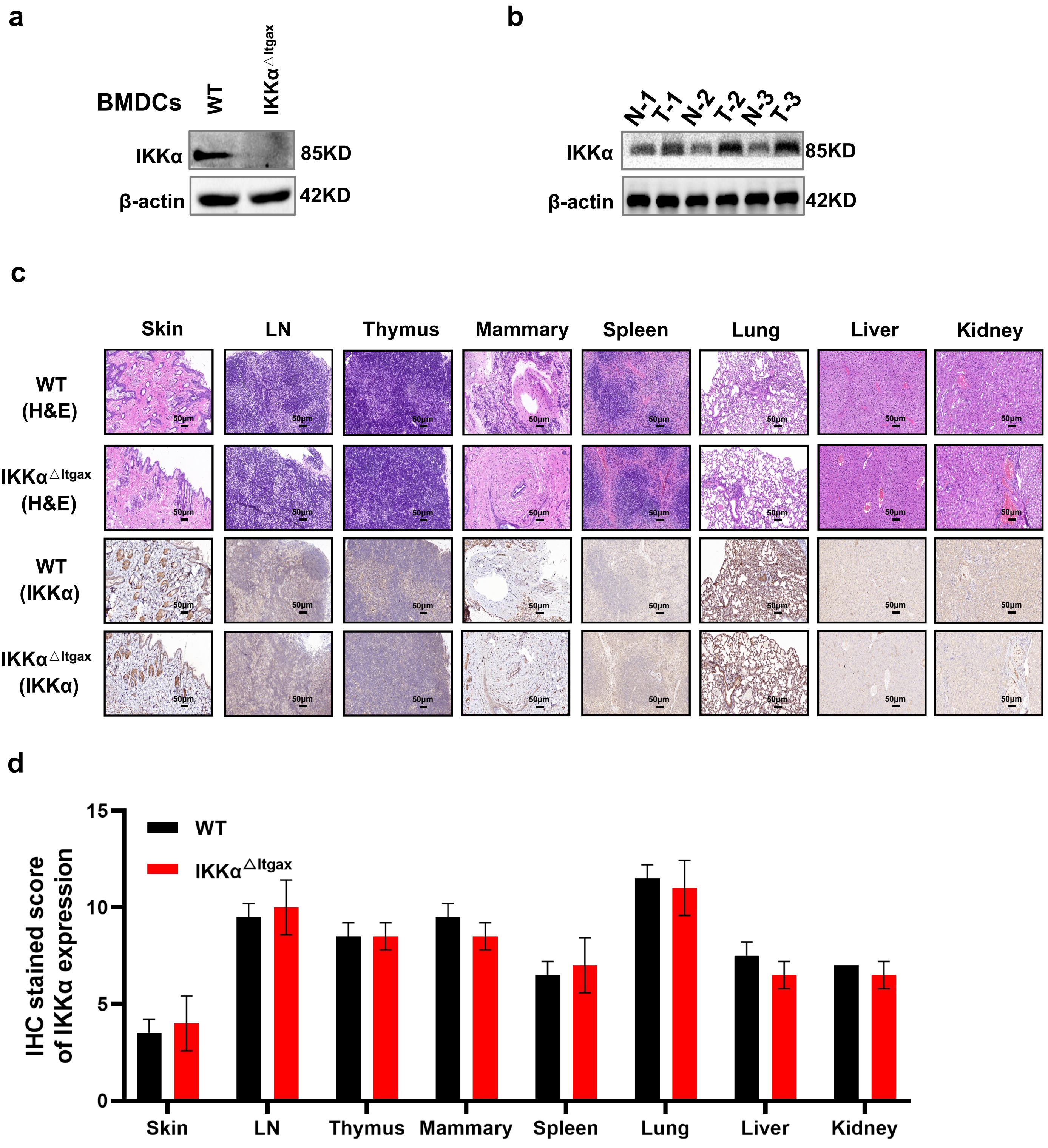
a
BMDCs
WT
IKKα△Itgax
IKKα
85KD
β-actin
42KD
b
N-1
T-1
N-2
T-2
N-3
T-3
IKKα
85KD
β-actin
42KD
c
Skin
LN
Thymus
Mammary
Spleen
Lung
Liver
Kidney
WT (H&E)
IKKα△Itgax (H&E)
WT (IKKα)
IKKα△Itgax (IKKα)
50μm
d
IHC stained score of IKKα expression
WT
IKKα△Itgax
0
5
10
15
Skin
LN
Thymus
Mammary
Spleen
Lung
Liver
Kidney

Figure S3

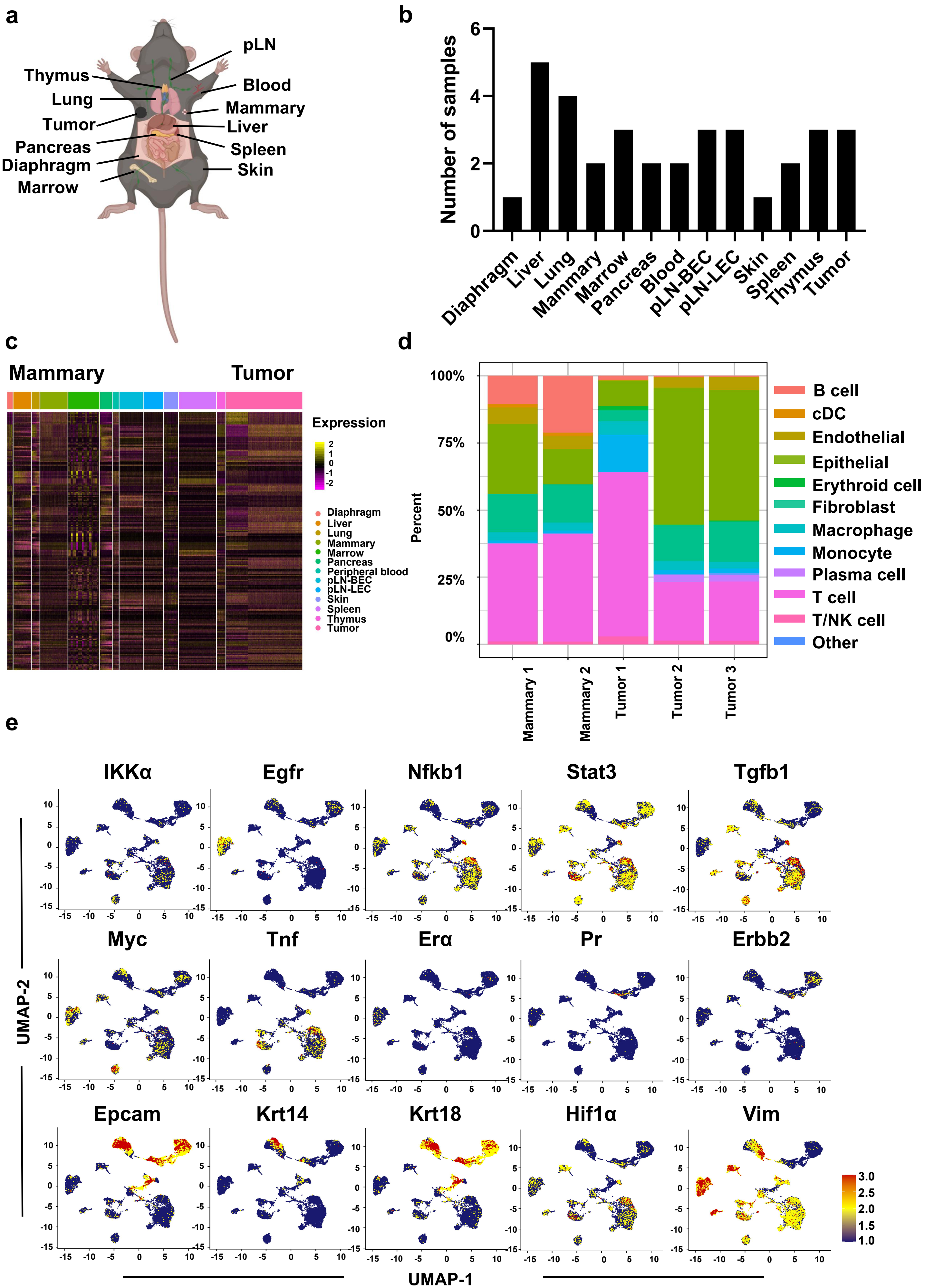
a
pLN
Thymus
Blood
Lung
Mammary
Tumor
Liver
Pancreas
Spleen
Diaphragm
Skin
Marrow
b
Number of samples
Diaphragm
Liver
Lung
Mammary
Marrow
Pancreas
Blood
pLN-BEC
pLN-LEC
Skin
Spleen
Thymus
Tumor
c
Mammary
Tumor
Expression
Diaphragm
Liver
Lung
Mammary
Marrow
Pancreas
Peripheral blood
pLN-BEC
pLN-LEC
Skin
Spleen
Thymus
Tumor
d
Percent
B cell
cDC
Endothelial
Epithelial
Erythroid cell
Fibroblast
Macrophage
Monocyte
Plasma cell
T cell
T/NK cell
Other
Mammary 1
Mammary 2
Tumor 1
Tumor 2
Tumor 3
e
IKKα
Egfr
Nfkb1
Stat3
Tgfb1
Myc
Tnf
Erα
Pr
Erbb2
Epcam
Krt14
Krt18
Hif1α
Vim
UMAP-2
UMAP-1

Figure S4

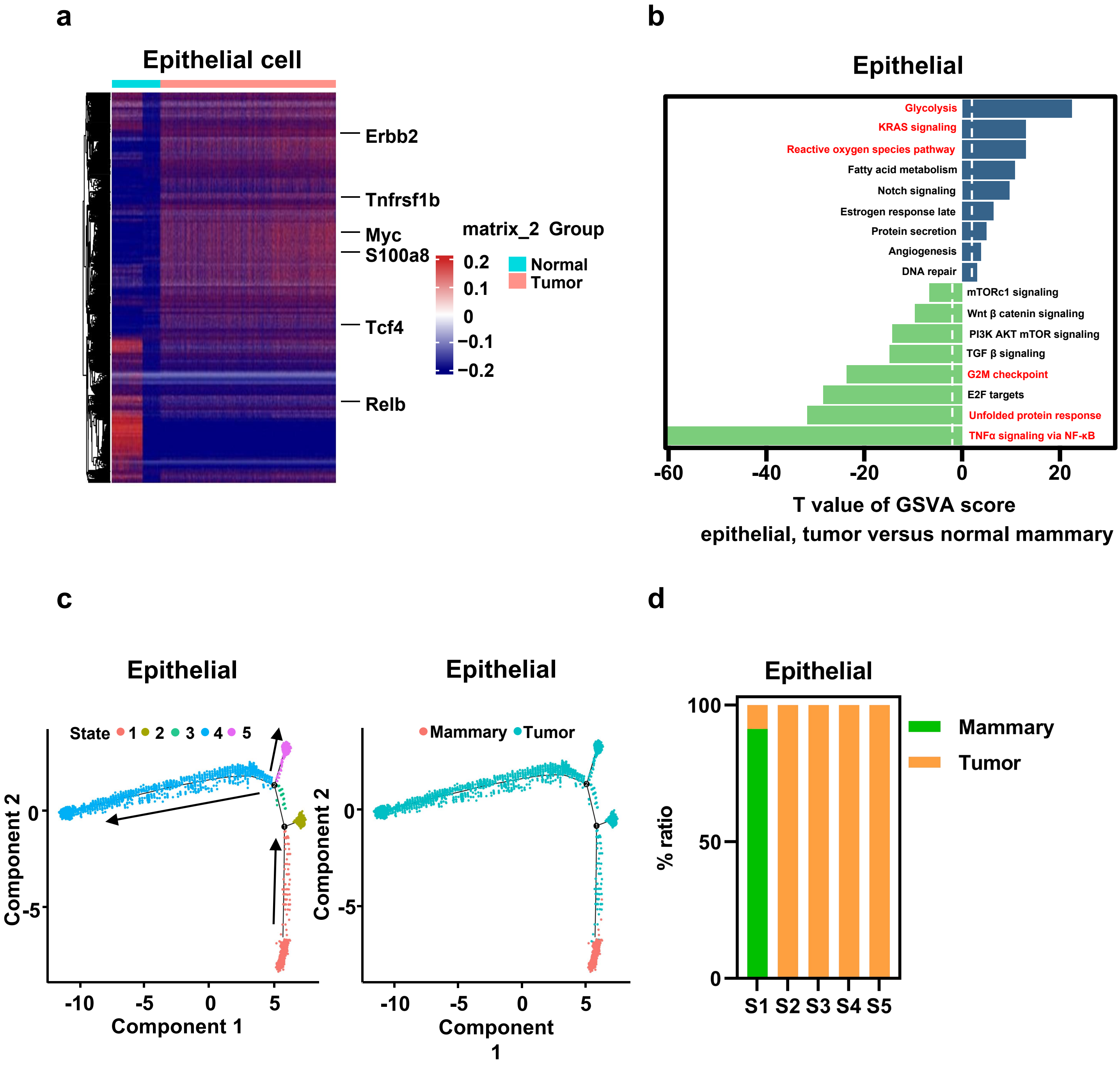
a
Epithelial cell
Erbb2
Tnfrsf1b
Myc
S100a8
Tcf4
Relb
matrix_2
0.2
0.1
0
−0.1
−0.2
Group
Normal
Tumor
b
Epithelial
Glycolysis
KRAS signaling
Reactive oxygen species pathway
Fatty acid metabolism
Notch signaling
Estrogen response late
Protein secretion
Angiogenesis
DNA repair
mTORc1 signaling
Wnt β catenin signaling
PI3K AKT mTOR signaling
TGF β signaling
G2M checkpoint
E2F targets
Unfolded protein response
TNFα signaling via NF-κB
-60
-40
-20
0
20
T value of GSVA score
epithelial, tumor versus normal mammary
c
Epithelial
State 1 2 3 4 5
Component 2
Component 1
-10
-5
0
5
Epithelial
Mammary Tumor
Component 2
Component 1
d
Epithelial
100
50
0
% ratio
Mammary
Tumor
S1 S2 S3 S4 S5

Figure S5

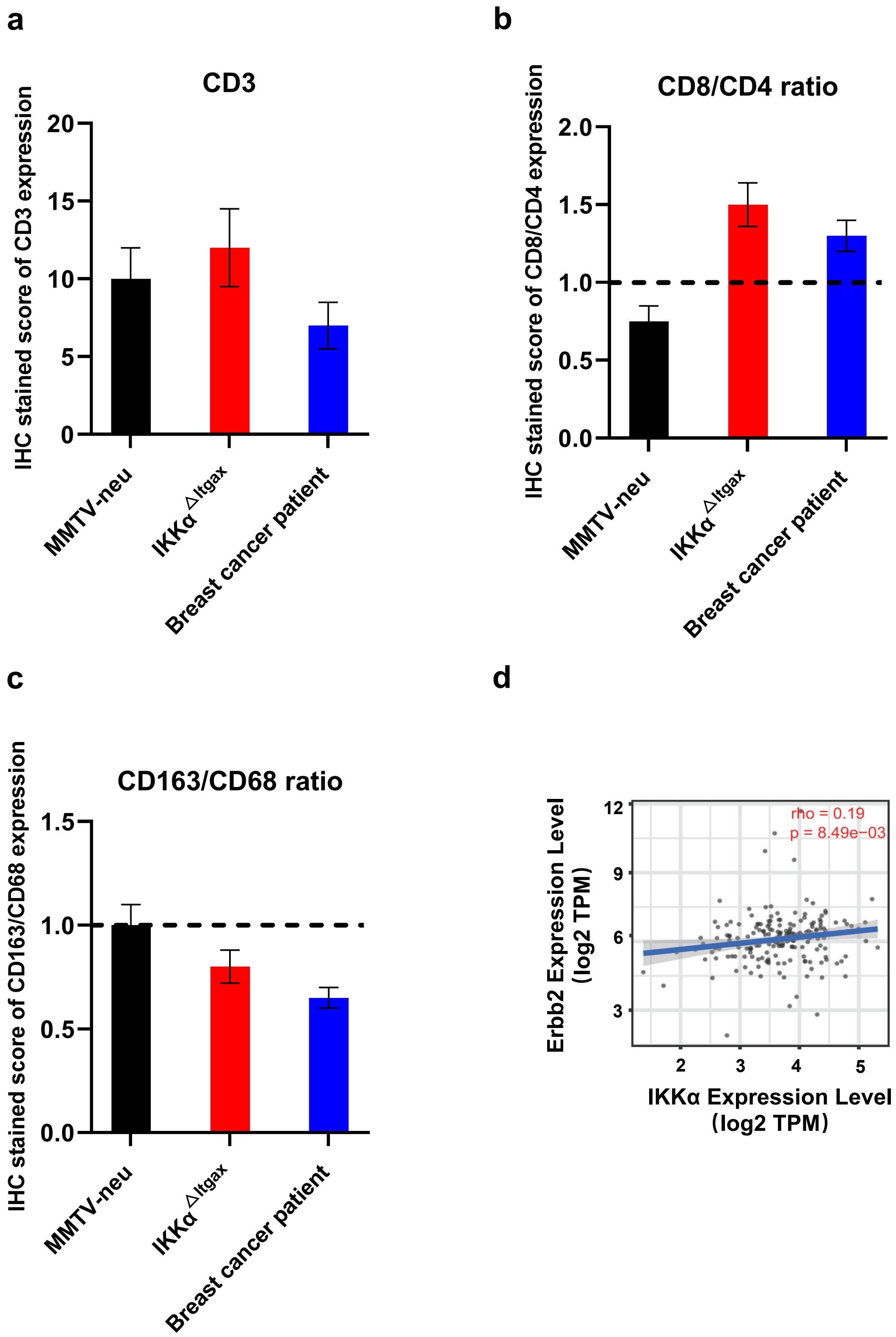
a
CD3
IHC stained score of CD3 expression
20
15
10
5
0
MMTV-neu
IKKα △Itgax
Breast cancer patient
b
CD8/CD4 ratio
IHC stained score of CD8/CD4 expression
2.0
1.5
1.0
0.5
0.0
MMTV-neu
IKKα △Itgax
Breast cancer patient
c
CD163/CD68 ratio
IHC stained score of CD163/CD68 expression
1.5
1.0
0.5
0.0
MMTV-neu
IKKα △Itgax
Breast cancer patient
d
rho = 0.19
p = 8.49e−03
Erbb2 Expression Level
(log2 TPM)
12
9
6
3
2
3
4
5
IKKα Expression Level
(log2 TPM)

Figure S6

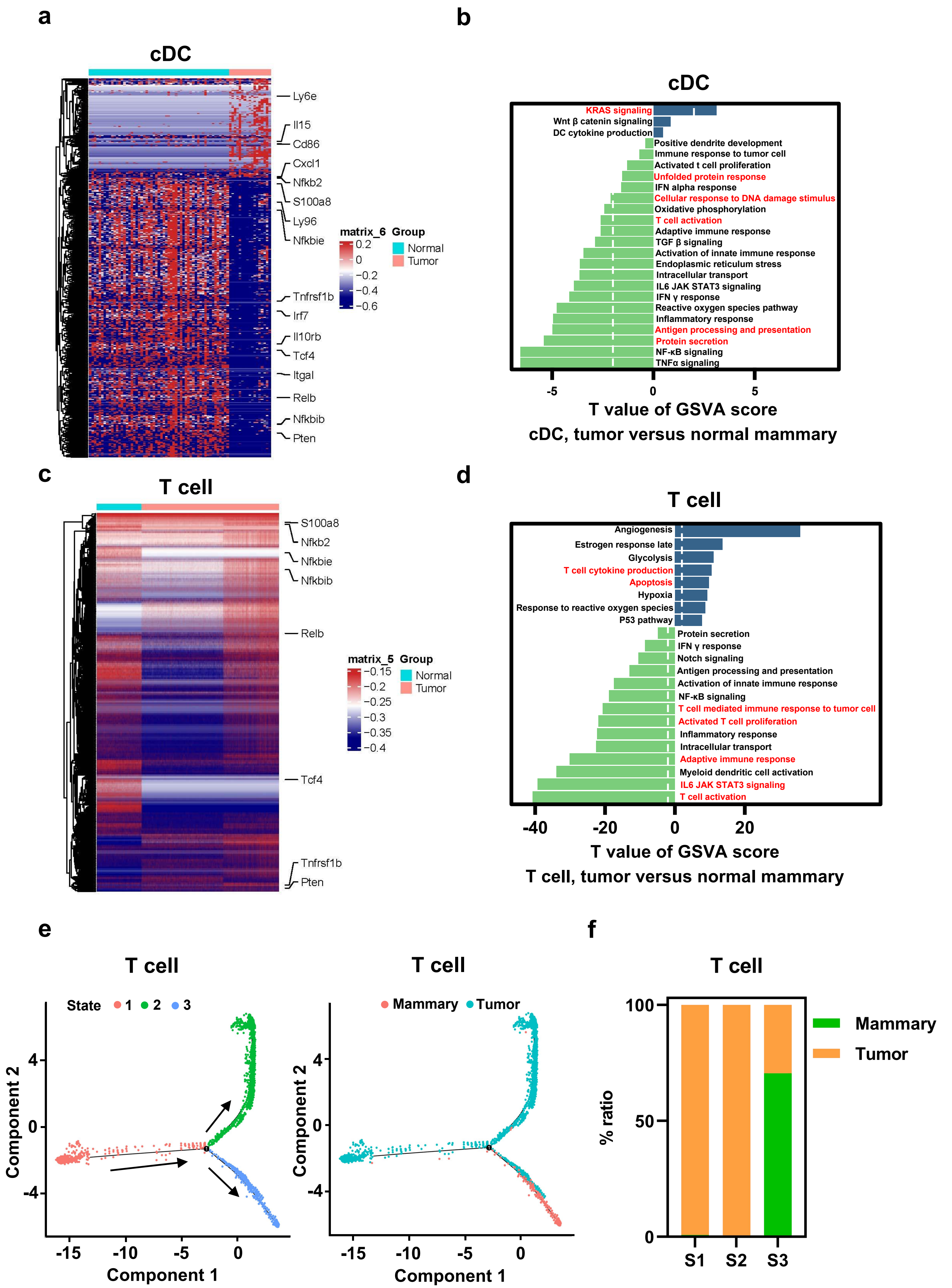

a
cDC
Ly6e
Il15
Cd86
Cxcl1
Nfkb2
S100a8
Ly96
Nfkbie
Tnfrsf1b
Irf7
Il10rb
Tcf4
Itgal
Relb
Nfkbib
Pten
matrix_6
0.2
0
−0.2
−0.4
−0.6
Group
Normal
Tumor
b
cDC
KRAS signaling
Wnt β catenin signaling
DC cytokine production
Positive dendrite development
Immune response to tumor cell
Activated t cell proliferation
Unfolded protein response
IFN alpha response
Cellular response to DNA damage stimulus
Oxidative phosphorylation
T cell activation
Adaptive immune response
TGF β signaling
Activation of innate immune response
Endoplasmic reticulum stress
Intracellular transport
IL6 JAK STAT3 signaling
IFN γ response
Reactive oxygen species pathway
Inflammatory response
Antigen processing and presentation
Protein secretion
NF-κB signaling
TNFα signaling
-5
0
5
T value of GSVA score
cDC, tumor versus normal mammary
c
T cell
S100a8
Nfkb2
Nfkbie
Nfkbib
Relb
Tcf4
Tnfrsf1b
Pten
matrix_5
−0.15
−0.2
−0.25
−0.3
−0.35
−0.4
Group
Normal
Tumor
d
T cell
Angiogenesis
Estrogen response late
Glycolysis
T cell cytokine production
Apoptosis
Hypoxia
Response to reactive oxygen species
P53 pathway
Protein secretion
IFN γ response
Notch signaling
Antigen processing and presentation
Activation of innate immune response
NF-κB signaling
T cell mediated immune response to tumor cell
Activated T cell proliferation
Inflammatory response
Intracellular transport
Adaptive immune response
Myeloid dendritic cell activation
IL6 JAK STAT3 signaling
T cell activation
-40
-20
0
20
T value of GSVA score
T cell, tumor versus normal mammary
e
T cell
State 1 2 3
Component 2
Component 1
4
0
-4
-15
-10
-5
0
T cell
Mammary Tumor
Component 2
Component 1
f
T cell
100
50
0
% ratio
S1
S2
S3
Mammary
Tumor

Figure S7

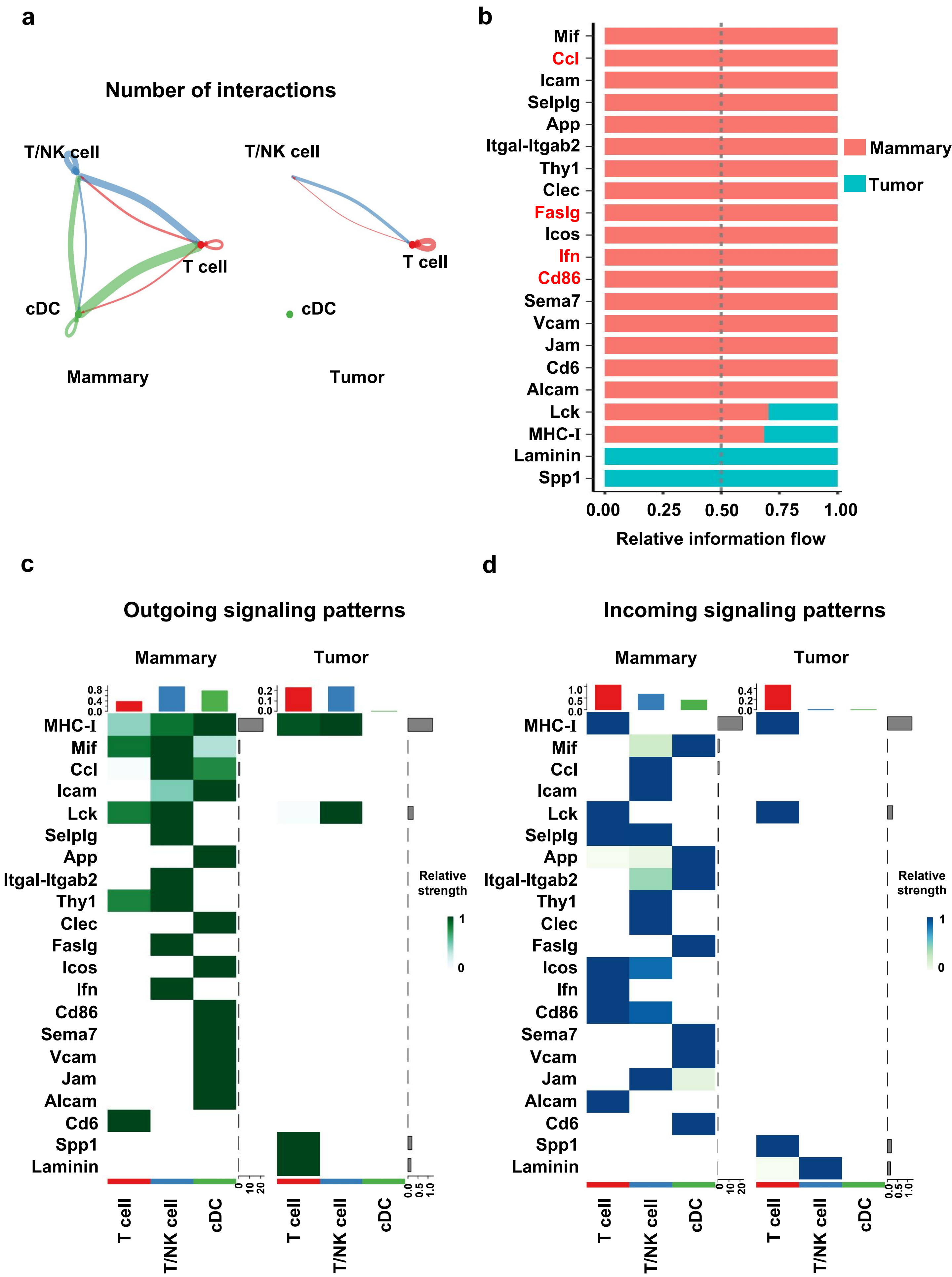

a
Number of interactions
T/NK cell
T cell
cDC
Mammary
T/NK cell
T cell
cDC
Tumor
b
Mif
Ccl
Icam
Selplg
App
Itgal-Itgab2
Thy1
Clec
Faslg
Icos
Ifn
Cd86
Sema7
Vcam
Jam
Cd6
Alcam
Lck
MHC-I
Laminin
Spp1
Mammary
Tumor
0.00
0.25
0.50
0.75
1.00
Relative information flow
c
Outgoing signaling patterns
Mammary
Tumor
MHC-I
Mif
Ccl
Icam
Lck
Selplg
App
Itgal-Itgab2
Thy1
Clec
Faslg
Icos
Ifn
Cd86
Sema7
Vcam
Jam
Alcam
Cd6
Spp1
Laminin
Relative strength
T cell
T/NK cell
cDC
T cell
T/NK cell
cDC
d
Incoming signaling patterns
Mammary
Tumor
Relative strength

Figure S8

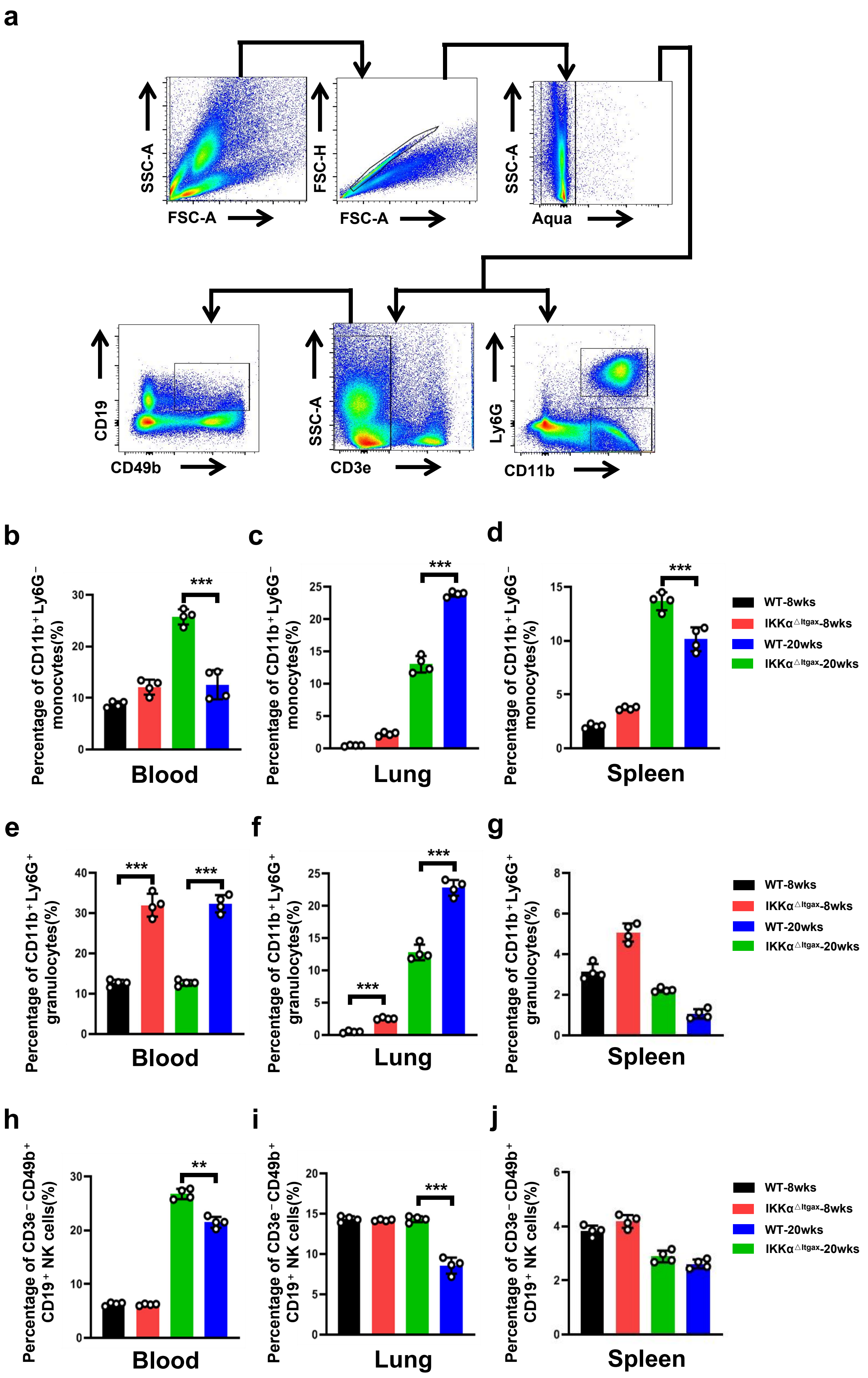

a
SSC-A
FSC-A
SSC-H
FSC-A
SSC-A
Aqua
CD19
CD49b
SSC-A
CD3e
Ly6G
CD11b
b
c
d
Percentage of CD11b⁺Ly6G⁻ monocytes(%)
***
Blood
Lung
Spleen
WT-8wks
IKKα△Itgax-8wks
WT-20wks
IKKα△Itgax-20wks
e
f
g
Percentage of CD11b⁺Ly6G⁺ granulocytes(%)
***
Blood
Lung
Spleen
WT-8wks
IKKα△Itgax-8wks
WT-20wks
IKKα△Itgax-20wks
h
i
j
Percentage of CD3e⁻CD49b⁺ CD19⁺ NK cells(%)
**
***
Blood
Lung
Spleen
WT-8wks
IKKα△Itgax-8wks
WT-20wks
IKKα△Itgax-20wks

Figure S9

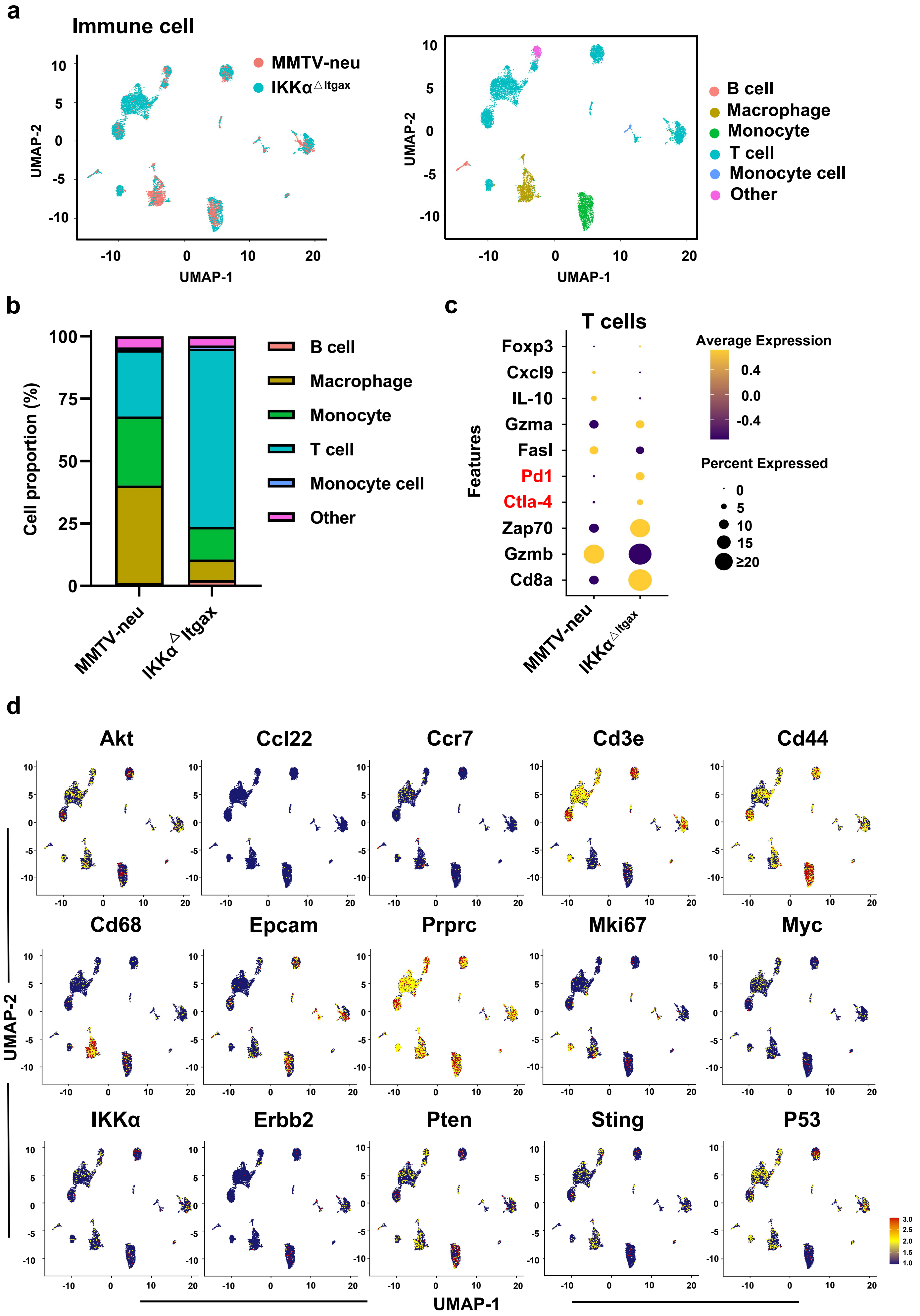

a
Immune cell
MMTV-neu
IKKα△Itgax
UMAP-1
UMAP-2
B cell
Macrophage
Monocyte
T cell
Monocyte cell
Other
b
Cell proportion (%)
MMTV-neu
IKKα△Itgax
c
T cells
Features
Foxp3
Cxcl9
IL-10
Gzma
Fasl
Pd1
Ctla-4
Zap70
Gzmb
Cd8a
Average Expression
Percent Expressed
d
Akt
Ccl22
Ccr7
Cd3e
Cd44
Cd68
Epcam
Prprc
Mki67
Myc
IKKα
Erbb2
Pten
Sting
P53

Figure S10

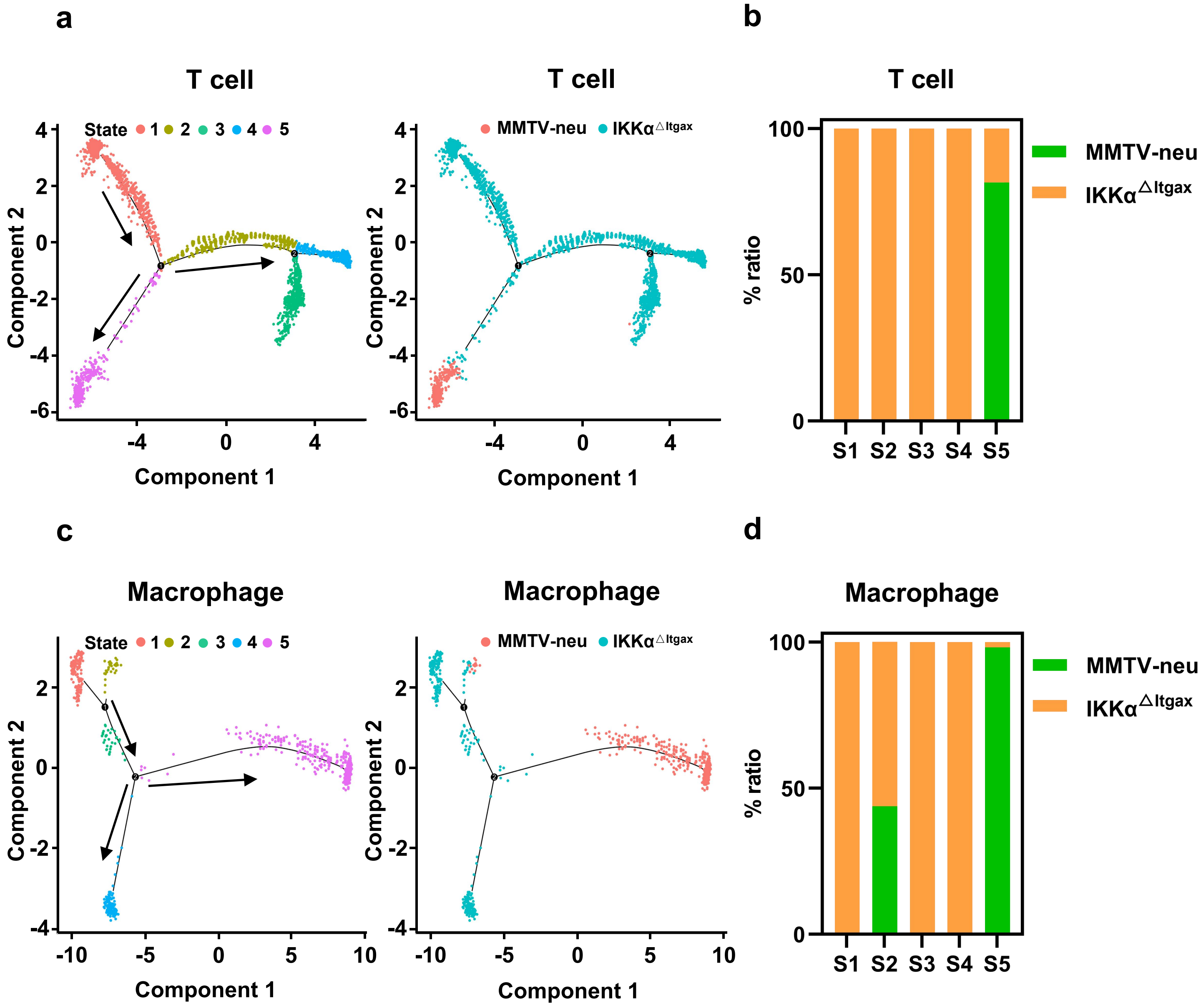
a
T cell
State 1 2 3 4 5
Component 2
Component 1
T cell
MMTV-neu IKKα△Itgax
Component 2
Component 1
b
T cell
MMTV-neu
IKKα△Itgax
% ratio
S1 S2 S3 S4 S5
c
Macrophage
State 1 2 3 4 5
Component 2
Component 1
Macrophage
MMTV-neu IKKα△Itgax
Component 2
Component 1
d
Macrophage
MMTV-neu
IKKα△Itgax
% ratio
S1 S2 S3 S4 S5
